# Transient expression of anti-HrpE scFv antibody reduces the hypersensitive response in non-host plant against bacterial phytopathogen *Xanthomonas citri* subsp. *citri*

**DOI:** 10.1038/s41598-024-57355-w

**Published:** 2024-03-26

**Authors:** Hamideh Raeisi, Mohammad Reza Safarnejad, Seyed Mehdi Alavi, Maxuel de Oliveira Andrade, Naser Farrokhi, Seyed Ali Elahinia

**Affiliations:** 1 Foodborne and Waterborne Diseases Research Center, Research Institute for Gastroenterology and Liver Diseases, Shahid Beheshti University of Medical Sciences, Shahid Arabi Ave., Yemen St., Velenjak, Tehran Iran; 2 Department of Plant Viruses, Agricultural Research Education and Extension Organization of Iran, Iranian Research Institute of Plant Protection, Tehran, Iran; 3 Department of Plant Biotechnology, National Institute of Genetic Engineering and Biotechnology, Tehran, Iran; 4 Brazilian Biorenewables National Laboratory (LNBR), Brazilian Center for Research in Energy and Materials (CNPEM), Campinas, Brazil; 5 Departement of Cell & Molecular Biology, Faculty of Life Sciences & Biotechnology, Shahid Beheshti University G.C, Evin, Tehran Iran; 6 Department of Plant Protection, College of Agricultural Sciences, Guilan University, Rasht, Iran

**Keywords:** Citrus canker, HrpE, Single chain fragment variable, Phage display, Transient expression, Bacteriology, Plant biotechnology, Biotechnology

## Abstract

Citrus canker is a bacterial disease caused by *Xanthomonas citri* subsp. *citri* (*Xcc*) that affects the citrus industry worldwide. Hrp pili subunits (HrpE), an essential component of Type III secretion system (T3SS) bacteria, play a crucial role in the pathogenesis of *Xcc* by transporting effector proteins into the host cell and causing canker symptoms. Therefore, development of antibodies that block HrpE can suppress disease progression. In this study, a specific scFv detecting HrpE was developed using phage display technique and characterized using sequencing, ELISA, Western blotting, and molecular docking. In addition, a plant expression vector of pCAMBIA-scFvH6 was constructed and agroinfiltrated into *Nicotiana tabacum* cv*. Samson* leaves. The hypersensitive response (HR) in the leaves of transformed and non-transformed plants was evaluated by inoculating leaves with *Xcc*. After three rounds of biopanning of the phage library, a specific human scFv antibody, named scFvH6, was identified that showed high binding activity against HrpE in ELISA and Western blotting. Molecular docking results showed that five intermolecular hydrogen bonds are involved in HrpE-scFvH6 interaction, confirming the specificity and high binding activity of scFvH6. Successful transient expression of pCAMBIA-scFvH6 in tobacco leaves was verified using immunoassay tests. The binding activity of plant-produced scFvH6 to detect HrpE in Western blotting and ELISA was similar to that of bacterial-produced scFvH6 antibody. Interestingly, tobacco plants expressing scFvH6 showed a remarkable reduction in HR induced by *Xcc* compared with control plants, so that incidence of necrotic lesions was significantly higher in non-transformed controls (≥ 1.5 lesions/cm^2^) than in the plants producing scFvH6 (≤ 0.5 lesions/cm^2^) after infiltration with *Xcc* inoculum. Our results revealed that the expression of scFvH6 in tobacco leaves can confer resistance to *Xcc*, indicating that this approach could be considered to provide resistance to citrus bacterial canker disease.

## Introduction

Citrus canker is one of the most economically important citrus diseases which is caused by *Xanthomonas citri* subsp. *citri* (*Xcc*)^1^. The bacterium causes destructive damage to several citrus cultivars by causing yellow chlorotic rings on leaves, stems, and fruits, inducing defoliation and fruit drop, and even reducing yield in severe cases^1,2^. Annually, millions of dollars have been spent to prevent and control citrus canker; however, the disease remains a major challenge with an undeniable economic impact on citrus production worldwide^3^. The bacteria use different strategies to successfully colonize host plants^4^. Type III secretion system (T3SS) is a key player in bacterial pathogenicity and acts through the injection of effector proteins into eukaryotic host cells^4,5^. T3SS is encoded by at least six operons of hrpA to hrpF, which are clustered in a 23-kb chromosomal region and play critical roles in the induction of hypersensitive response and bacterial pathogenicity^5^. T3SS translocates the bacterial effector protein into the cytosol of the host cell through an extracellular filamentous appendage, termed Hrp pilus, which acts as a channel between bacterial and plant cells^6^. Hrp pili have been characterized in T3SS-containing plant pathogens^6,7^. The structural component required for pilus formation in *Xanthomonas* is HrpE protein, which is a 9.7 kDa protein and shows high conservation among *Xanthomonas* strains, including *Xcc, X. oryzae* pv. *oryzae*, *X. axonopodis* pv. *glycines*, *X. campestris* pv. *campestris*, and *X*. *campestris* pv. *vesicatoria*^6^. In these bacteria, the assembly of HrpE into the pilus is essential for the secretion of different transcription activator-like (TAL) effectors, which play key roles in *Xanthomonas*-host interactions^6^. Therefore, HrpE exerts a critical role in bacterial pathogenicity due to transporting TAL effectors such as PthA into cytosol plants. The effector protein PthA is the most important factor in the production of canker symptoms in host plants^8^. So far, many methods have been attempted to control the disease using copper-based antimicrobials, uprooting and burning infected citrus trees, traditional breeding to generate canker-resistant citrus varieties, and transgenic expression of resistance genes and antimicrobial agents (AMPs)^1,9^. Citrus plants produced by transgenic overexpression and CRISPR genome editing approaches have shown promising results in conferring disease resistance^10,11^; however, only a few genetic engineering studies have shown enhanced canker resistance^12^. Recently, transgene-free canker-resistant citrus cultivars have been generated via Cas/gRNA ribonucleoprotein (RNP) complex editing of the coding region of canker susceptibility gene citrus sinensis lateral organ boundary 1 (CsLOB1), resulting in enabling canker resistance^13^.

Over the last three decades, the expression of recombinant proteins in plant cells against specific plant pathogens has received much attention as a new strategy to minimize losses and produce resistant plants^14,15^. Recombinant-antibody-mediated resistance in plants was first reported in the early 1990s^16^, and since then, different recombinant antibodies, e.g., fragment antigen-binding region (Fab), single chain variable fragments (scFvs), and single-domain antibody (sdAbs), have been expressed in plants against different plant pathogens^17–19^. Among the various forms of recombinant antibodies, scFv is the most applicable type, which retains antigen specificity and function of the full-size antibody^20^. Phage display is the most conducive technology for the development of recombinant scFv fragments. This technology screens libraries of random peptides to isolate fragments with high specificity and sensitivity and provides high-quality tests that are less time-consuming^21^. So far, this strategy has been used for plant expression of scFv antibodies against different targets^17,22–24^.

Some studies have demonstrated that the HrpE mutant of *Xanthomonas* was deficient in promoting disease symptoms in the host plants, indicating the critical role of HrpE in bacterial pathogenicity^25^. In addition, it was shown that HrpE can elicit plant immune response and hypersensitivity in non-host plants^26^. In support of these findings, HrpE could be a candidate for producing resistant plants through plant antibodies. In addition, because of HrpE conservation, anti-HrpE scFvs may recognize HrpE from different strains of *Xanthomonas*, resulting in broad-spectrum resistance.

We previously designed the specific primers from HrpE sequence submitted in GenBank under Acc. No. WP_011050291.1; and amplified HrpE gene (282 bp) from an Iranian A* isolate of *Xcc* (NIGEB-088). The PCR product was cloned into pTG-T19 vector, followed by subcloning into pET28a (+) bacterial expression vector bearing an N-terminal 6× His tag sequence. The recombinant protein of HrpE (rHrpE) was expressed in *Escherichia coli* strain Rosetta (DE3), and the integrity of the expressed protein was confirmed by Western blot analysis^27^.

**Figure 1:**
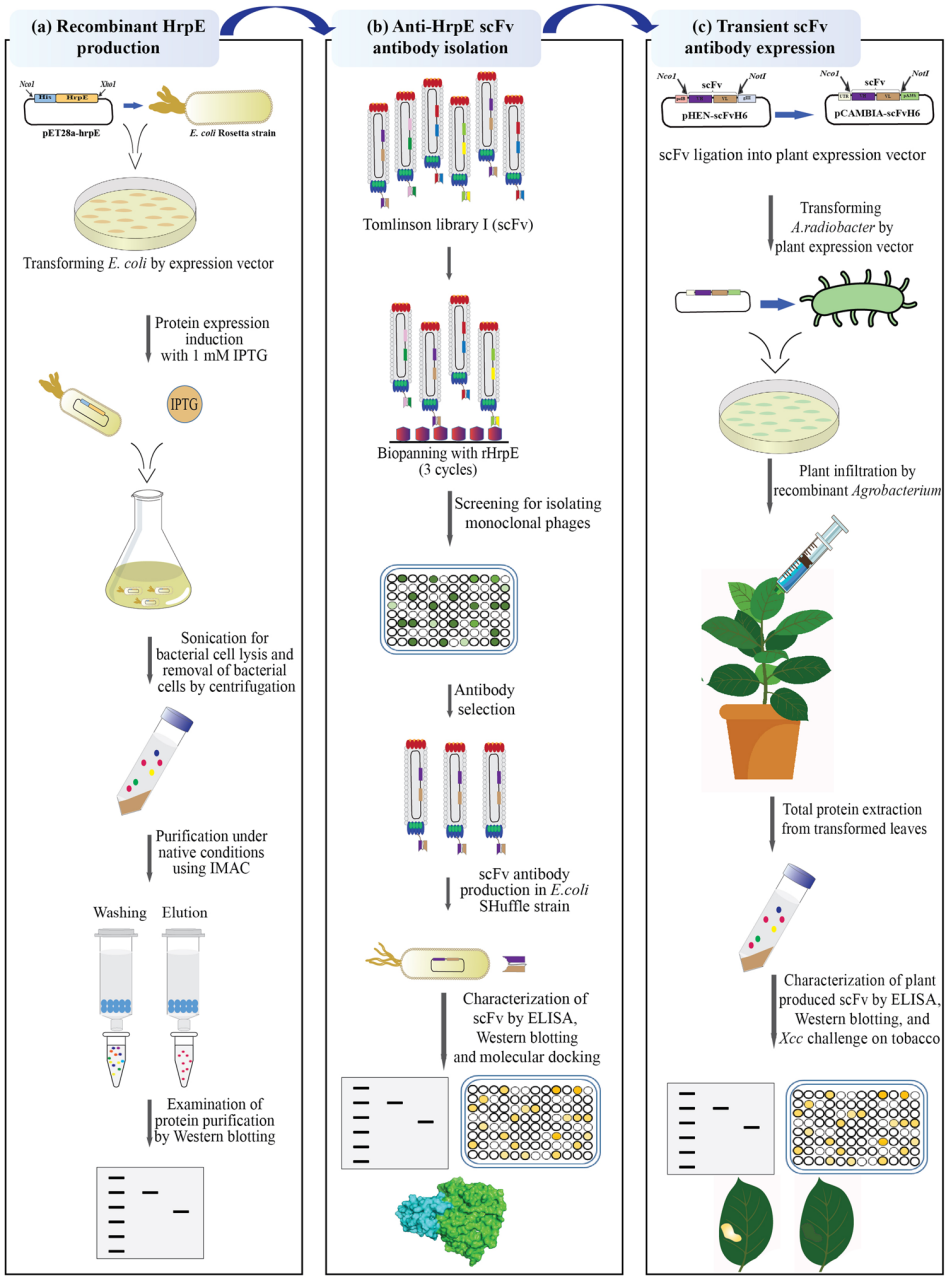
A schematic diagram of the experimental setup used in this study. (**a**) Representation of expression and purification of rHrpE, (**b**) selection of monoclonal scFv antibody from phage display library and characterization of selected scFv (scFvH6) against HrpE, (**c**) transient expression of scFvH6 antibody in tobacco plant model and characterization of plant-produced scFvH6 against HrpE at in vitro and in vivo levels. *A. radiobacter*: *Agrobacterium radiobacter*; ELISA: enzyme-linked immunosorbent assay; *E. coli: Escherichia coli*; IMAC: immobilized-metal affinity chromatography; IPTG: isopropyl ß-D-1-thiogalactopyranoside; rHrpE: recombinant HrpE; scFv: single-chain variable fragment; *Xcc*: *Xanthomonas citri* subsp. *citri.*

In this study, we described an optimized protocol for generating highly sensitive and specific scFv detecting rHrpE using phage display technology. The binding activity of selected scFv to detect rHrpE was assessed by ELISA, Western blotting, and molecular docking. Furthermore, we promoted *Nicotiana tabacum* cv *Samson* transient expression of a specific anti-HrpE scFv antibody and examined the functional activity of plant-produced scFv against rHrpE and *Xcc*. An overview of the study design is shown in Fig. 1. This study provides insights into the development of *Xcc*-resistant plants by introducing anti-HrpE scFv antibody gene into citrus cultivars.

## Results

### Enrichment of phages with specific binding to rHrpE

**Figure 2:**
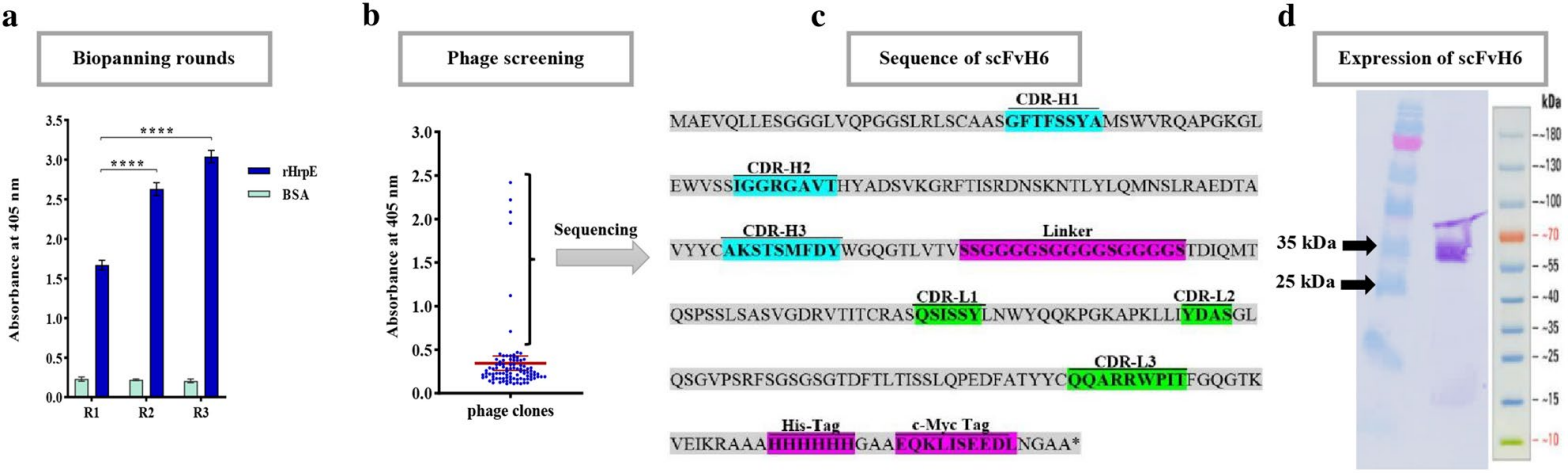
(**a**) Enrichment of Tomlinson I scFv library against rHrpE through three rounds of biopanning. (**b**) Phage-ELISA for screening of 94 individual phage clones obtained from the third round of panning against rHrpE. (**c**) The amino acid sequences of selected scFv antibody (scFvH6) with binding specificity to rHrpE (including depiction of CDRs, His-tag and c-Myc tag). (**d**) Western blot analysis to assay purification of scFH6 using anti-His tag antibody (1:10,000). BSA was considered as negative control. Data are represented as mean ± SD from three independent experiments. ****P < 0.0001, significantly different from control. BSA: bovine serum albumin; CDRs: complementarity-determining regions; M: Marker PageRuler Prestained Protein Ladder (Thermo Scientific, USA); rHrpE: recombinant HrpE.

To identify specific scFvs targeting HrpE, firstly, recombinant protein of HrpE was expressed in *E. coli* Rosetta strain. The results of SDS-PAGE and Western blotting showed the correct expression and purification of rHrpE at the expected size of 15 kDa with a concentration of about 700 µg/mL (Supplemental Fig. 1). To select antibodies against rHrpE, the human scFv library I was used. Antibodies were selected in microtiter plates against rHrpE in three rounds of biopanning. Based on the results, an increase in the phage recovery rate occurred during three rounds of selection, as far as an increase of 750-fold in the phage recovery rate of the final biopanning round was observed compared with the first round (Table 1). Monitoring the progression of biopanning rounds using polyclonal phage ELISA confirmed that the specificity of eluted phage displaying scFvs against rHrpE was significantly increased after three rounds of biopanning. There was no reaction between the phages obtained from biopanning rounds with BSA as a negative control (*P* < 0.0001) (Fig. 2a).

**Table 1:** Titers of phage libraries I eluted after biopanning processes against rHrpE.

Round of panning	Input phage	Output phage
1	10^13^	7.2 × 10^4^
2	10^13^	2.5 × 10^6^
3	10^13^	5.4 × 10^7^

rHrpE: recombinant HrpE.

### Identification of a specific scFv antibody against recombinant HrpE

The output obtained after three rounds of biopanning was transformed into *E. coli* TG1 strain. The ability of 94 single clones to bind to rHrpE was determined by phage-ELISA. Based on the results, six enriched phage clones showed the highest signals for detecting rHrpE (Fig. 2b). The analysis of variations within the genes coding for scFv fragments using *Bst*NI showed that all scFv genes had similar restriction endonuclease profiles (Supplemental Fig. 2), indicating the same sequence for all positive clones selected in phage-ELISA. This result was confirmed by DNA sequencing analysis of scFvs with high binding activity, revealing a distinct scFv sequence containing 6 complementarity-determining regions (CDRs): CDRHI-CDRHIII at positions 28–35, 53–60, and 99–107 and CDRLI-CDRLIII at positions 161–166, 184–187, and 223–231, respectively. This scFv was named scFvH6 and submitted to GenBank under Acc. No. OR513891. The complete gene sequence of scFvH6 is shown in Fig. 2c.

### In vitro binding activity and cross-reactivity of scFvH6 antibody against HrpE

**Figure 3:**
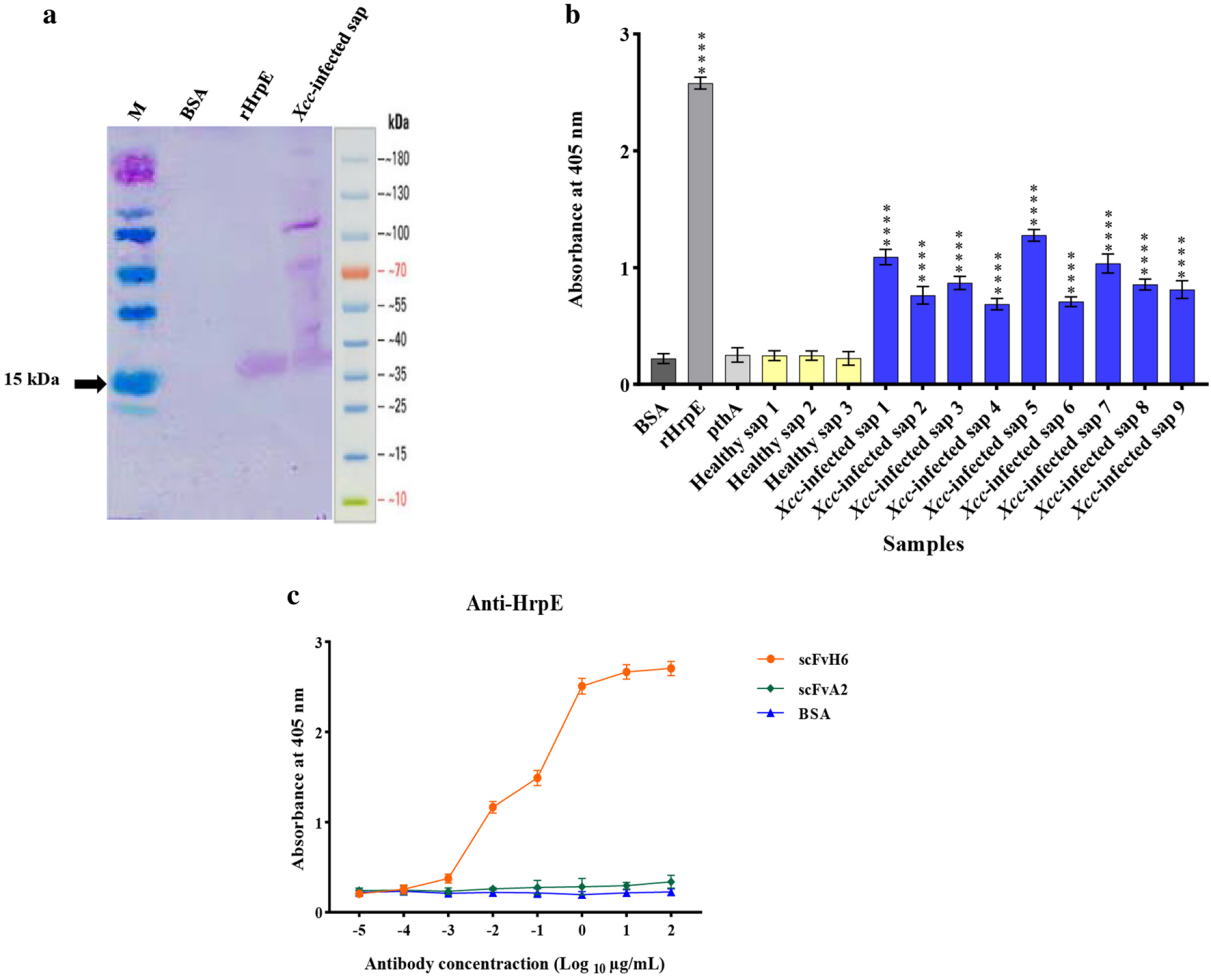
Characterization of in vitro binding activity of scFvH6 against rHrpE and native HrpE in *Xcc*-infected samples using (**a**) Western blot analysis and (**b**) indirect ELISA. (**c**) Titration ELISA to detect the working concentration of scFvH6. BSA was used as negative control. Data are represented as mean ± SD from three independent experiments. ****P < 0.0001, significantly different from control. BSA: bovine serum albumin; M: Marker PageRule Prestained Protein Ladder (Thermo Scientific, USA); rHrpE: recombinant HrpE; *Xcc*: *Xanthomonas citri* subsp. *citri*.

To characterize the efficiency of scFvH6 antibody against HrpE, we first expressed scFv in SHuffle strain of *E. coli* at the expected size (approximately 32 kDa) with yields about 420 µg/mL (Fig. 2d). Analysis of the specificity of scFvH6 by Western blotting exhibited a single band for rHrpE and native HrpE protein at the expected size of 15 kDa, whereas there was no reaction with BSA, demonstrating that scFvH6 specifically bound to HrpE (Fig. 3a).

To confirm the ability of scFvH6 to bind to native HrpE protein, indirect ELISA was carried out using *Xcc*-infected samples. The results showed that the highest absorption rate was observed for rHrpE protein (10 µg/mL), followed by samples one, five, and seven of *Xcc*-infected samples. The rate of adsorption of scFvH6 in all infected samples was more than twice those of the wells containing healthy samples. Absorbance of PthA was similar to BSA as the negative control, confirming non-cross-reactivity with other proteins (Fig. 3b).

To further characterize the binding activity of scFvH6 antibody to rHrpE, the ability of different concentrations of scFvH6 to detect rHrpE was checked by ELISA titration. The results demonstrated that scFvH6 detected rHrpE in a concentration-dependent manner, so that the lowest concentration detecting rHrpE was about 0.01 µg/mL (Fig. 3c).

### In silico analysis of scFvH6 antibody affinity for HrpE

**Figure 4:**
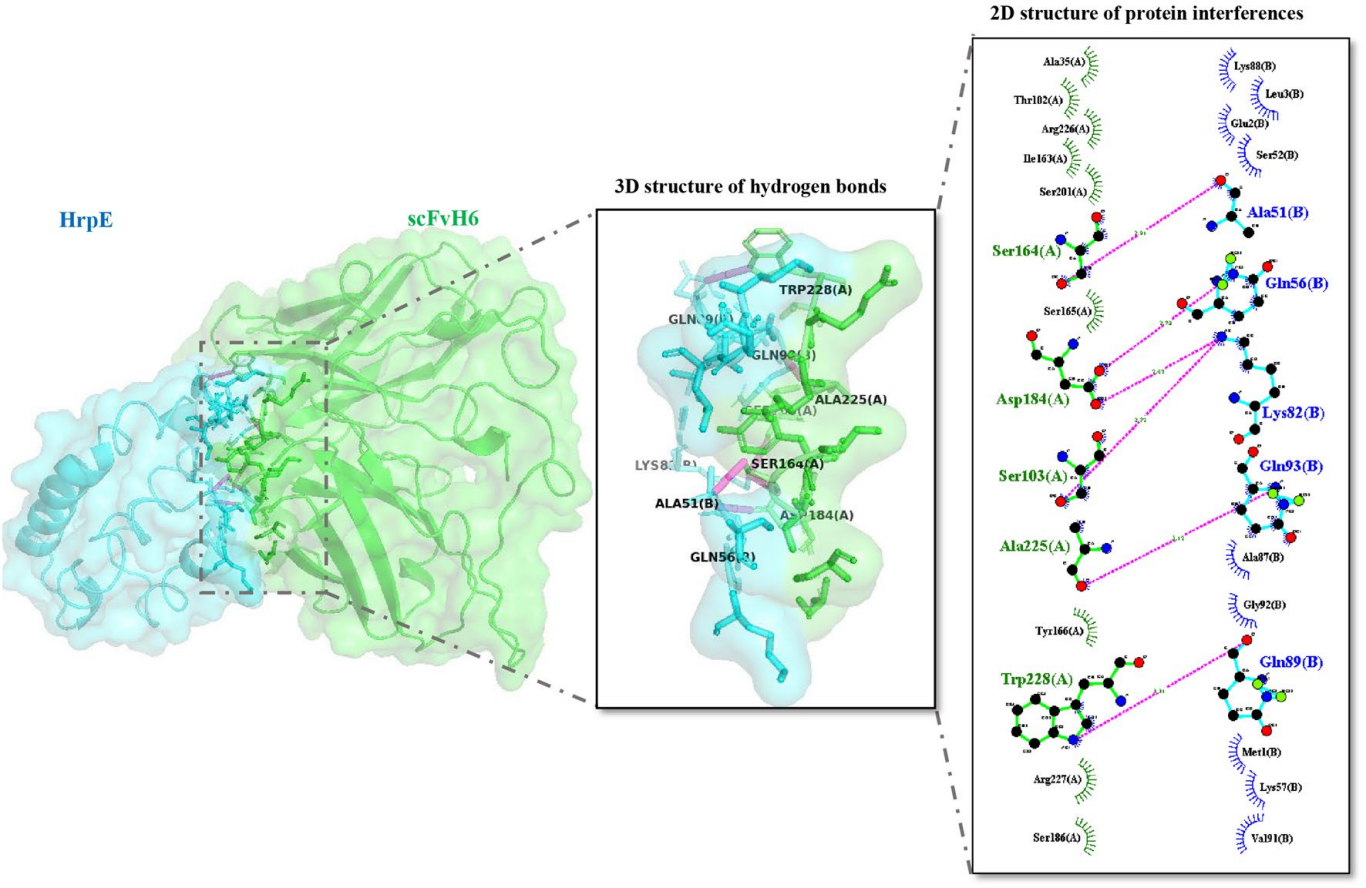
3D structure presentation of the binding poses of HrpE (blue) and scFvH6 (green). Hydrogen bonds involved in scFvH6-HrpE interaction are shown in the 3D structure as pink lines, and total intermolecular analyses containing hydrogen bonds and Van der Waals forces of the interaction are also presented in 2D structure (A: antibody and B: antigen). 2D: two-dimensional; 3D: three-dimensional.

To investigate the structural basis of the interaction between scFvH6 and HrpE, the crystal structure of scFv was determined using I-TASSER server service. The Z-score predicted by ProSA indicated high-quality models (Supplemental Fig. 3). The results of docking outputs demonstrated a large binding interface between scFvH6 and HrpE, which was primarily comprising CDRH3, CDRL1, CDRL2, and CDRL3 of scFv fragment (Table 2). This interaction involved five hydrogen bonds and nine hydrophobic interactions. Consistently, most of the protein interferences were mediated by the light chain, so that 10 residues from scFvH6 light chain interacted with HrpE (Fig. 4).

**Table 2:** Amino acid residues of scFvH6 involved in the hydrogen bonds with rHrpE.

CDR	Position	
	Heavy chain	Light chain
1	–	Ser: 164
2	–	Asp: 184
3	Ser: 103	Ala: 225, Trp: 228

CDRs: complementarity-determining regions; rHrpE: recombinant HrpE.

### Expression of scFvH6 antibody fragments in plant leaves

**Figure 5:**
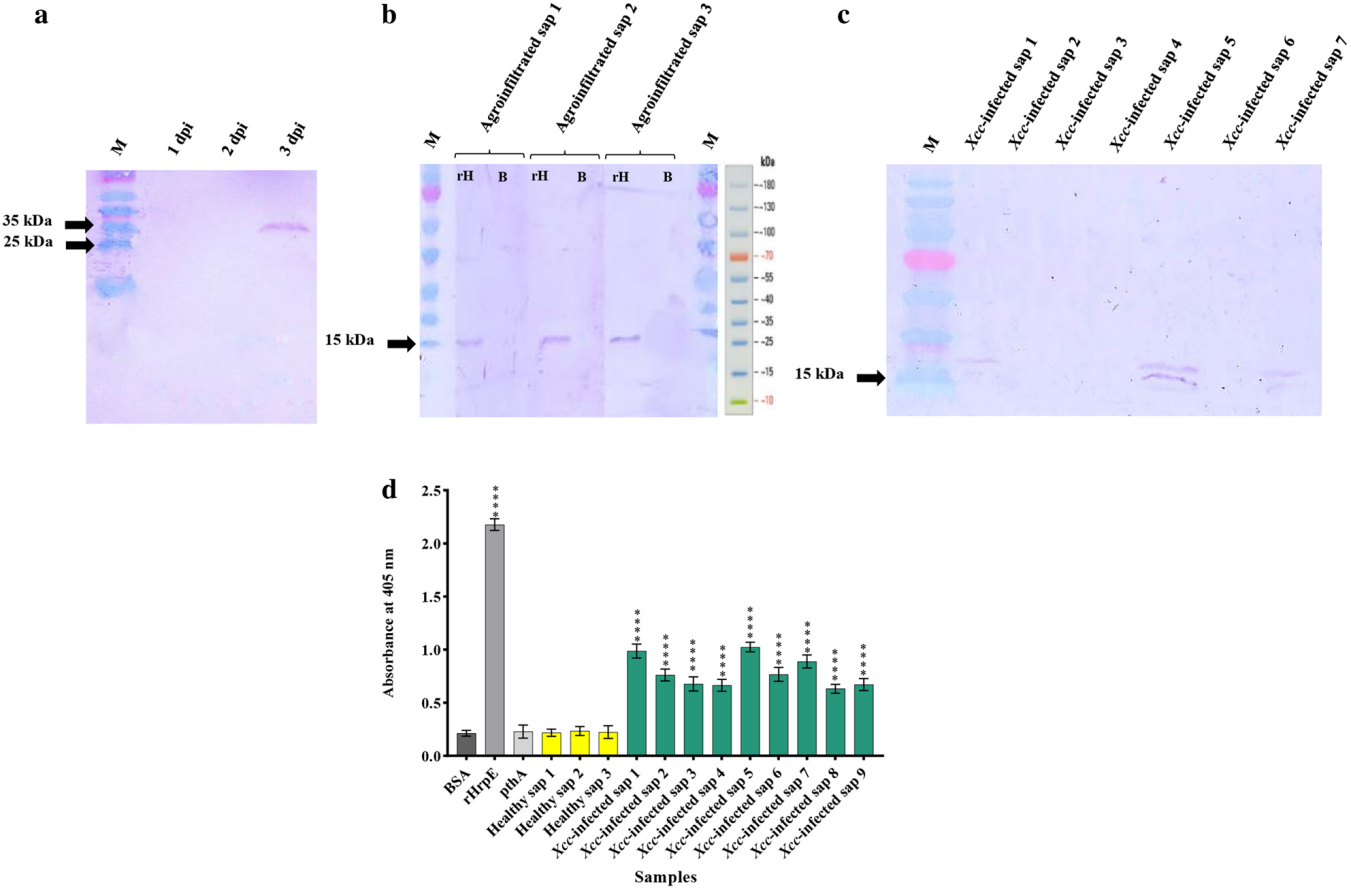
Detection and characterization of expression of scFvH6 in leaves of *Nicotiana tabacum* cv*. Samson*. (**a**) Temporal analysis of the expression scFvH6 in transformed leaves during 1–3 days post inoculation (dpi) using Western blotting. (**b**) The binding activity of scFvH6 expressed in different transformed leaves to detect rHrpE using Western blotting. (**c**) The binding activity of plant-produced scFvH6 against rHrpE and native HrpE in *Xcc*-infected samples using Western blotting and (**d**) indirect ELISA. Data are represented as mean ± SD from three independent experiments. ****P < 0.0001, significantly different from control. B: bovine serum albumin; M: Marker PageRuler Prestained Protein Ladder (Thermo Scientific, USA); rH: recombinant HrpE; *Xcc*: *Xanthomonas citri* subsp. *citri.*

To study the expression of scFvGH6 antibody in *N. tabacum* cv*. Samson* leaves, pCAMBIA-scFvH6 cassette was constructed and transformed into *Agrobacterium radiobacter*. The transformed agrobacteria were recognized by PCR using scFv-specific primers (supplemental Fig. 4) and infiltrated into tobacco leaves for transient expression. Temporal analysis of the expression and the integrity of scFvH6 in infiltrated leaves using Western blotting showed a detectable expression level of scFvH6 at the expected size (approximately 35 kDa) after 3 day post-inoculation (dpi), indicating the correct expression and folding of scFvH6 in plant cells (Fig. 5a). Therefore, this time point was selected to take plant material for total protein extraction.

### In vitro binding activity of plant-produced scFvH6 to HrpE

To determine functionality of the plant-produced scFvH6 antibody, the total proteins of the transformed plant were extracted and used as a primary antibody in immunoassay tests. Western blotting results showed that total proteins extracted from different agroinfiltrated leaves detected rHrpE at the expected size of 15 kDa (Fig. 5b). Additionally, the native form of HrpE was detected with plant-derived scFv antibody at the expected size in samples one, five, and seven of *Xcc*-infected plants (Fig. 5c).

In a parallel experiment, the results of indirect ELISA demonstrated that plant-produced scFvH6 detected rHrpE and *Xcc*-infected plants, whereas no reaction with healthy and negative control samples was found. Intestinally, samples one, five, and seven of *Xcc*-infected plants that were detected in Western blotting displayed the highest absorbance in ELISA among other infected samples, revealing differences in concentration of HrpE protein among the samples. These results showed that plant-produced scFvH6 is functional and strongly binds to HrpE (Fig. 5d). The binding activity of plant-derived scFvH6 antibody to HrpE was further compared with that of scFvH6 produced in bacterial cells. As expected, scFvH6 produced in both plant material and bacterial cells displayed similar binding properties (Supplemental Fig. 5).

### Function of scFvH6 expressed in tobacco leaves to limit hypersensitive response activated by *Xcc*

To study the specificity of scFvH6, leaves of *N. tabacum* cv. *Samson* was initially inoculated with *Xcc*, PBS, and agrobacterium bearing pCAMBIA-scFvH6. Based on our results, necrotic lesions developed in leaves infiltrated by *Xcc*, indicating that *Xcc* induced HR in *N. tabacum* cv. *Samson.* Notably, the distinct necrotic lesions induced by *Xcc* inoculation first developed 2 dpi, whereas the controls, i.e., PBS and agrobacterium bearing pCAMBIA-scFvH6, were nearly symptomless (Supplemental Fig. 6a). Symptoms continued to develop through 3-day evaluation period. Notably, the size of the necrotic lesions developed by *Xcc* in tobacco leaves was approximately 1.5 cm^2^ after 3 dpi, whereas necrotic lesions were not considerable in treatment with pCAMBIA-scFvH6 or PBS (Supplemental Fig. 6b).

**Figure 6:**
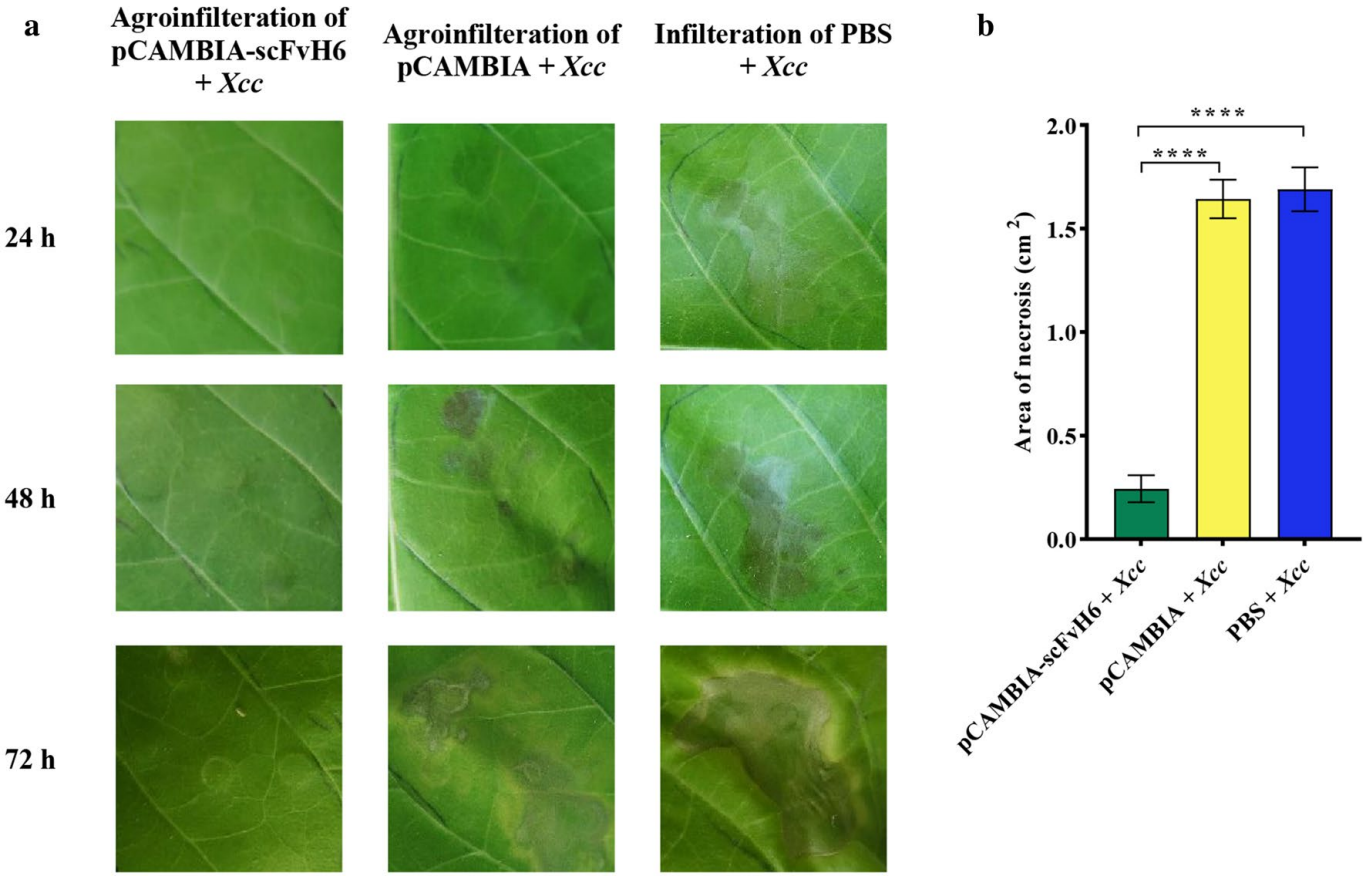
(**a**) Appearance of *N. tabacum* leaves infiltrated with agrobacterium bearing pCAMBIA-scFvH6w, agrobacterium bearing vector control (pCAMBIA1300), and PBS that after 3 days were inoculated with suspension of *Xcc* (10^8^ CFU/mL). (**b**) Comparison of the size of the necrotic lesions developed by *Xcc* in different treatments (2 dpi). Data are represented as mean ± SD from six independent experiments. ****P < 0.0001, significantly different from control. PBS: phosphate-buffered saline; *Xcc*: *Xanthomonas citri* subsp. *citri.*

Subsequently, when the leaves were agroinfiltrated with pCAMBIA-scFvH6 and inoculated with *Xcc* 3 days later, HR was suppressed and necrotic lesions arising from *Xcc* were significantly limited compared with controls (Fig. 6a). Agroinfiltration of leaves with pCAMBIA (as vector control) and infiltration with PBS did not affect necrotic lesions arising from *Xcc*, so that necrotic area in these treatments was larger than 1.5 cm^2^ (Fig. 6b). These results showed that scFvH6 functionally blocked HrpE activity and interrupted the development of hypersensitive response in tobacco leaves against *Xcc*.

## Discussion

The use of antibody fragments to confer resistance has been an effective approach against some plant diseases^15,28,29^. In these strategies, producing antibodies in plant cells can interfere in pathogenesis of pathogens and develop resistance to them^28^. Recently, the role of HrpE in the secretion of bacterial effector proteins has been characterized^6^. Accordingly, because of the crucial role of HrpE in *Xcc* pathogenesis, the present study was conducted to develop and characterize a specific phage display-derived scFv targeting HrpE. Moreover, transient expression of the anti-HrpE scFv antibody in *N. tabacum* cv. *Samson* leaves was assessed. Our results demonstrated that a unique scFv antibody, scFvH6, was selected after three rounds of biopanning, showing high binding activity against HrpE even at low concentrations (> 0.01 µg/mL). The high sensitivity and binding activity of specific scFvs to detect various antigens have been reported in different studies^23,30^. Additionally, the Western blotting result showed a single band at the expected size to detect rHrpE, indicating high specificity of scFvH6. Previous studies have demonstrated that scFv antibodies can be reliable tools with high specificity and sensitivity to detect different antigens^17,22,23,31,32^, which is in accordance with our results. Additionally, our previous work revealed that the use of specific scFvs against components of T3SS of *Xcc* can show high specificity for detecting *Xcc* infection in plants^18,33^. The results of molecular docking showed that four CDRs (CDRH3, CDRL1, CDRL2, and CDRL3) of scFvH6 were involved in the interaction with HrpE epitopes by forming five intermolecular hydrogen bonds. These interactions demonstrated a specific and strong binding of scFvH6 to HrpE and may explain the high binding activity of scFvH6 in immunoassay tests. Several studies have demonstrated that each of the six CDRs can be involved in antibody-antigen interactions; however, the involvement of CDR3 in antigen interactions has been mostly reported, which is in agreement with our results^18,23,34^.

Previous studies have demonstrated that transient gene expression in plants allows the study of in vivo protein function^35,36^. Agrobacterium infiltration is a widely used method for transient expression studies in plants and provides a cost-effective procedure for studying gene function^36^. It is well documented that the infiltration of plant leaves with agrobacterium bearing scFv genes can confer resistance to different pathogens^18,24,32^. In addition, we previously showed that transient expression of anti-PthA scFv antibody suppressed HR activated by *Xcc* in the plant model^18^. Different studies have discussed the critical role of HrpE in the pathogenesis of *Xanthomonas*. These studies have demonstrated that the HrpE mutant of *Xanthomonas* could not elicit HR in a non-host plant and pathogenicity in a host plant. Interestingly, this mutant failed to translocate effector proteins into host cells, resulting in a complete loss of *Xanthomonas* pathogenicity^6,37^. Supporting the role of HrpE in translocating effectors to plant cells, the transient expression of scFvH6 in non-host *N. tabacum* cv. *Samson* leaves was done to evaluate the inhibitory activity of this antibody on HR triggered by *Xcc*. Notably, tobacco is widely used for transient or stable gene expression, because it can be easily transformed by *A. radiobacter*^35,38^. Since *Xcc* can elicit HR at the site of infection in tobacco leaves, transient expression of scFvH6 in tobacco provides a short-term test to verify in vivo antibody function. Based on our results, agroinfiltration of *N. tabacum* cv*. Samson* leaves with pCAMBIA-scFvH6 construct led to the expression of scFvH6 in leaves at a detectable level after 3 dpi, which was relatively in agreement with other studies^17,18,32^. Notably, contrarily to these studies, non-detection of scFv protein in Western blotting has been a major challenge in other studies, which may be due to low-abundance antibody in plant cells^17^; however, several studies have demonstrated that plant-produced antibodies can confer resistance to specific pathogens even at low levels^17,18,24,39^. In our study, scFvH6 produced in plants could detect HrpE in immunoassay tests, supporting its correct expression and protein folding. Interestingly, both scFvH6 produced in bacteria and plant cells exhibited similar binding activities against rHrpE and cognate HrpE, which was in accordance with previous studies^18,24,32^.

Remarkably, the challenge of tobacco leaves expressing scFvH6 antibodies with *Xcc* significantly reduced the development of *Xcc*-induced necrotic lesions compared with the control plants non-expressing scFvH6, further confirming that the functional expression of anti-HrpE scFv antibody in plant cells and its inhibitory activity on T3SS of *Xcc*. These findings may explain the function of HrpE in the translocation of effector proteins from bacterial cells to plant cells^6^. The stability and functionality of scFv antibodies during transient expression in plants have been reported in other studies^17,24,32^.

Stable expression of recombinant antibodies in transgenic plants has been reported previously^40^. Transgenic plants producing specific scFvs confer resistance to different plant diseases^19,28,39,41^. Although there was no report about stable expression of scFvs to control *Xcc*, different genetic engineering approaches have been used to obtain *Xcc*-resistant plants^10,12,13^*.* The development of transgenic plants expressing antimicrobial peptides, resistance genes, and immune-related genes has been reported to increase canker resistance^12^. The use of CRISPR/Cas-mediated genome editing of the promoter or coding region of LOB1 has also introduced to confer citrus resistance to *Xcc*^10,42^. However, the use of transgenic plants may face concerns regarding food and environmental safety^43^. Recently, the use of the Cas/gRNA RNP complex to generate transgene-free crops has received much attention. This method presents an efficient transgene-free genome-editing strategy for conferring resistance to citrus *Xcc*^13^. The main limitation of our study was that the expression of scFvH6 was only estimated in the plant model, and we didn’t assess the scFv expression in host plants. However, it should be noted that agrobacterium infiltration is not used widely in citrus due to its low efficiency, therefore, there is a need for a more robust transient expression system in citrus leaves^44,45^. Additionally, the stable expression of scFvH6 in plants can help prove the ability of this antibody to control *Xcc*, which requires a time- and labor-intensive procedure and may not be provided by agrobacterium-mediated transformation^46^. Hence, our findings can provide preliminary insight into the development of more tolerant plants to *Xcc* in the future.

In conclusion, we successfully isolated a potent scFv antibody targeting HrpE of *Xcc* using the phage display technique, named scFvH6, which showed high binding activity in different in vitro assays and in silico analysis. Additionally, transient expression of scFvH6 in non-host tobacco leaves suppressed *Xcc-*triggering HR by inhibiting HrpE, probably because of the inability to transfer effector proteins to plant cells. The present study for the first time provides evidence for the transient expression of scFv targeting HrpE in a plant model; thus, further research is required to determine interventions of scFvs on HrpE activities in other *Xanthomonas* strains using stable expression in host plants.

## Materials and methods

### Plant materials and growth conditions

Leaf and stem samples from lime plants with blister-like lesions were collected from the southern regions of Iran, and their *Xcc* infection status was checked by PCR using hrpE-specific primers (Table 3, Supplemental Fig. 7). Additionally, *N. tabacum* cv *Samson* were cultivated in an insect-free greenhouse at 22 °C, 80% relative humidity, with a 16:8 h light: dark photoperiod.

**Table 3:** Oligonucleotide sequences used in this study.

Target gene	Oligonucleotide sequence (5′–3′)	Products (bp)	T_m_ ºC	Reference
HrpE	F: GTCGACAAATGGAATTATTACCG R: CTCGAATTACTGGCCAACGGCTG	282	61	^27^
pHEN	F: GCCGCTGGATTGTTATTACCTCT R: CTATGCGGCCCCATTCA	850	54	This work
ScFv	F: ATCCTTCGCAAGACCCTTCCTCT R: AGAGAGAGATAGATTTGTAGAGA	900	57	^18^

### Extraction of total proteins from plant samples

Extraction of plant proteins was conducted as described previously^33^. Briefly, plant leaves were ground to powder using liquid nitrogen, homogenized, and extracted in two volumes of extraction buffer (5 mM EDTA, 5 mM β-mercaptoethanol, 1× PBS, pH 7.5). Leaf extracts were vigorously vortexed and then centrifuged at 10,000×*g* for 15 min at 4 °C. The supernatants were filtered through 0.45 µm pore filters (Sartorius, Germany).

### Production of HrpE recombinant protein

The HrpE gene of NIGEB-088 (Acc. No. GCF_002742455.1), an Iranian A* isolate of *Xcc*, was previously cloned into pET28a (+) with N-terminally 6× His tag^27^. To induce protein expression, *E. coli* Rosetta strain was transformed by pET28-HrpE plasmid and cultured in 2× YT broth (16 g/L tryptone, 10 g/L yeast extract, 5 g/L NaCl, pH 7.2) supplemented with 50 µg/mL kanamycin and 0.1% (w/v) glucose. Expression of protein was induced with 1 mM isopropyl ß-d-1-thiogalactopyranoside (IPTG) at OD600 = 0.4; and then the culture was incubated at 28 °C overnight. After that, the cells were harvested by centrifugation (10,000×*g*, 4 °C, 20 min) and lysed using sonication at 50% amplitude 10 s pulse settings for 3–5 min. Protein purification was performed using immobilized-metal affinity chromatography (IMAC) (Qiagen, Germany) under native conditions using buffers containing 20–250 mM imidazole gradient. Purified protein was separated on 12% SDS-PAGE and transferred to a polyvinylidene fluoride (PVDF) membrane (Sigma-Aldrich, Germany). The membrane was blocked with 5% skimmed milk powder (Fluka, Germany) and then incubated with anti-His tag antibody (1:10,000) (Abcam, UK), followed by alkaline phosphatase (AP) conjugated anti-mouse antibody (1:7000) (Sigma-Aldrich, Germany). Protein signals were detected using the substrate 5-bromo-4-chloro-3-indolyl phosphate (BCIP) and nitro-blue tetrazolium (NBT) (Sigma-Aldrich, Germany). Protein concentration was estimated by measuring the sample absorbance at 280 nm.

### Enrichment of phage display library against recombinant HpE

To select specific scFv antibodies, biopanning rounds were conducted on Tomlinson I scFv phage library (MRC, Cambridge, England, ReIn_0017), as described previously^31^. Briefly, an immunotube (Nunc Inc, Denmark) was coated with rHrpE (~ 100 µg/mL) and kept at 4 °C for 16 h. The coated immuno-tube was blocked with 3% (w/v) bovine serum albumin (BSA) (Fluka, Neu-Ulm, Germany), washed with 1× PBS, and incubated with phage suspension (~ 10^13^ cfu) to allow phage-protein binding at 37 °C for 2 h. After incubation, the immuno-tube was rinsed with PBST (1× PBS with 0.1% Tween-20) to remove unbound phages. The elution of antigen-bound phages was carried out using 100 mM trimethylamine, and the pH was neutralized by adding 1 M Tris–HCl (pH = 7.4). The eluted phages were amplified by reinfection of *E. coli* TG1 cells at OD600 = 0.4, and amplified phages were rescued from bacterial cells by superinfection with M13KO7 helper phage (about 10^9^ pfu/mL). The amplified phages were used in the next round of biopanning. The recovery rate of eluted phages was determined after each round of biopanning^23^.

### Specificity assessment of phage clones for detection of recombinant HpE

To assess the enrichment of antigen-specific clones, polyclonal phage-ELISA was conducted after each biopanning round as described previously^31^. Briefly, ELISA plates were coated with rHrpE (10 µg/mL), blocked with 3% skimmed milk powder, and washed with PBST. Eluted phages obtained from each round of biopanning were added to 96-well microtiter plates and incubated at 37 °C for 2 h. After washing with PBST, the plates were incubated with an anti-M13-HRP conjugate antibody (1:10,000) (Abcam, UK) at 37 °C for 2 h. The reactions were detected using 2,2-azino-di-3-ethylbenz-thiazoline sulfonate (ABST) (Ferramentas, Lithuania) substrate. After stopping the reaction by adding 100 μL 1 N H_2_SO_4_, the absorbance was measured at 405 nm using an ELISA plate reader (Tecan, Switzerland). The reactions were considered positive when the absorbance of the analyzed samples was more than twice the absorbance of BSA (3%).

### Identification of specific recombinant phages for HrpE

To isolate monoclonal anti-HrpE binders, phages were further screened in a 96-well microtiter plate as described previously^22^. Briefly, *E. coli* TG1 cells were infected with phages obtained from the last round of biopanning. Bacterial cells bearing phage particles were plated on TYE medium agar supplemented with 1% (w/v) glucose and 100 µg/mL ampicillin. Individual colonies (94) were randomly picked and cultured in 96-well microtiter plates containing 2 × YT with 1% (w/v) glucose and 100 µg/mL ampicillin. Bacterial culture was superinfected with M13KO7 helper phage (about 10^9^ pfu/mL) to rescue phage clones from bacterial cells and then incubated at 30 °C for 16 h. After incubation time, the culture supernatants containing the individual phage clones were separated by centrifugation (2000×*g*, 4 °C, 10 min) and used as primary antibodies to detect rHrpE in phage-ELISAs. Binding specificity of phages was assessed as described in polyclonal phage ELISA.

### Characterization of scFvs antibodies

#### Endonuclease digestion and sequence analysis

The clones detecting rHrpE were amplified using pHEN primers designed by Oligo software version 7 (Table 3). The amplicons were digested with *Bst*NI (Ferramentas, Lithuania) and separated using agarose gel electrophoresis (2% w/v) as described by Pansri et al.^47^. In addition, Sanger sequencing (Pishgam Biotech Co., Tehran, Iran) was done for positive clones using pHEN primers. The sequence alignment analysis was performed using IMGT/V-QUEST alignment tool (http://www.imgt.org).

### Western blotting

To express scFv fragments, the phagemids were transformed into *E. coli* SHuffle strain. Protein expression was induced with 1 mM IPTG at OD600 = 0.4–0.6; and bacterial culture was incubated at 30 °C overnight. The specific scFv antibody was purified using IMAC under native conditions. Protein purification was analyzed using SDS-PAGE and Western blotting using anti-His tag antibody and AP-conjugated anti-mouse IgG antibody. Target proteins were visualized by adding BCIP/NBT substrate. Antibody concentration was determined by measuring absorbance at 280 nm.

To evaluate the ability of scFv antibody to detect antigen, rHrpE (10 μg/mL), total soluble proteins from healthy and *Xcc*-infected plant leaves (5 μL), and BSA as a negative control were separated on 12% SDS-PAGE; and transferred to PVDF membranes. The protein-transferred membranes were incubated with 1 µg/mL scFv as a primary antibody at 4 °C for 16 h. Target proteins were detected as described earlier.

### Enzyme-linked immunosorbent assay (ELISA)

To assess specificity of the scFv antibody, indirect ELISA was used as previously described^23^. Briefly, 96-well microtiter plates were coated with healthy and *Xcc*-infected plant extracts, rHrpE (10 µg/mL) as a positive control, and BSA as a negative control; and kept at 4 °C for 16 h. Additionally, recombinant protein pthA of *Xcc* previously prepared by^28^ was used as an antigen for detection of cross-reactivity of selected scFvs. After blocking, wells were incubated with 1 µg/mL scFv antibody at 4 °C for 16 h, followed by an anti-c-myc tag antibody (1:5000) (Abcam, USA) at room temperature for 2 h. The bound scFv antibody was detected using goat-anti-mouse HRP secondary antibody (1:7000) (Abcam, UK), and colorimetric detection was performed by adding ABST substrate, as described earlier.

Additionally, to determine scFv antibody titer, a titration ELISA was performed^23^. To do this, a dilution series of the scFv antibody (0.00001, 0.0001, 0.001, 0.01, 0.1, 1, 10, 100 μg/mL) was prepared and used to detect rHrpE (10 μg/mL). scFvA2 antibody that exhibited very low absorption in phage-ELISA and BSA were considered as negative controls. The antibody-antigen reaction was detected as described earlier. The lowest concentration of scFv antibodies that detected rHrp was considered as the working concentration.

### Homology modeling and molecular docking

To further characterize the binding activity of the selected scFv to HrpE, the interaction between these proteins was evaluated in silico. To do this, the tertiary structures (3D) of HrpE and scFvH6 were modeled using I-TASSER server service (https://zhanglab.ccmb.med.umich.edu/I-TASSER/). The predicted 3D structural models were refined by using ModRefiner server service (http://zhanglab.ccmb.med.umich.edu/ModRefiner/) to select structures closer to the native structure. The accuracy of the generated homology models was assessed using ProSA server service (https://prosa.services.came.sbg.ac.at/prosa.php). The mode of interaction between HrpE and scFvH6 was investigated using Haddock server service (https://haddock.science.uu.nl/services/HADDOCK2.2/). The selection of model was conducted based on the minimal local energy and the largest cluster size. The possible interactions between antibody and antigen were visualized using Pymol software version 1.5.0.1 (http://pymol.findmysoft.com). A detailed analysis of the visualization and 2D structural representations was accomplished using Ligplot plus software version (4.5.3).

### ScFv-mediated inhibition of HrpE in vivo

#### scFv cloning into plant expression vector

The DNA sequence encoding anti-HrpE scFvH6 was obtained by digesting scFvH6 construct derived from scFv phage display library by *Nco*I/*Not*I restriction enzymes and ligated in pCAMBIA1300 plant expression vector under the control of 35S *cauliflower mosaic virus* (CaMV) promoter with *Tobacco etch virus* 5′ untranslated region (UTR), which previously prepared by Raeisi et al.^18^. The new vector, pCAMBIA-scFvH6, was transformed into *A. radiobacter* GV3101 strain (gentamicin, kanamycin, and rifampicin resistance) by heat shock method. The transformed agrobacteria were checked by PCR using scFv-specific primers (Table 3).

### Transient expression of anti-HrpE scFv antibody in tobacco leaves

Transformed *A. radiobacter* was cultured in Luria broth (LB) medium supplemented with 10 mg/L rifampicin and 50 mg/L kanamycin at 28 °C with shaking at 150 rpm. Bacterial culture at OD_600_ = 1 were centrifuged (4300×*g*, 10 min, 4 °C); and bacterial cells were resuspended in agroinfiltration buffer (0.43% MS salts, 10 mM MES, 2% (w/v) sucrose, 200 mM acetosyringone, pH5.6) to an OD600 of 0.5 and incubated at room temperature for 3 h. Agroinfiltration of 6-week-old *N. tabacum* cv*. samson* plants was carried out by injecting a suspension of recombinant agrobacteria into the lower epidermis of intact leaves using a syringe without a needle. The suspension of *Xcc* (10^8^ CFU/mL of NIGEB-088 solution in 1× PBS) and 1× PBS buffer were infiltrated into tobacco leaves as controls. After 3 days, agroinfiltrated leaf material was harvested and total soluble proteins were extracted using two volumes of extraction buffer. Western blotting was done to evaluate scFvH6 in leaf total proteins at different time points as previously described. All experiments were performed using six leaves of six independent biological replicates.

### Characterization of plant-produced scFv antibody

To estimate functional ability of produced antibody in plant to detect *Xcc*, Western blot and indirect ELISA were done^18^. To do this, the total protein of the transgenic plant was extracted as previously described and used as a primary antibody to detect rHrpE (10 µg/mL) and native HrpE in protein extracts from *Xcc*-infected samples. Extracted proteins from healthy plants and BSA were considered as negative controls. The reactions were detected as described earlier.

### scFvH6 transient expression in tobacco leaves and hypersensitive response evaluation against *Xanthomonas citri* subsp. *citri* (*Xcc*)

To evaluate the ability of plant-produced scFvH6 to reduce *Xcc* symptoms, recombinant agrobacteria bearing pCAMBIA-scFvH6 were infiltrated into the *N. tabacum* cv. *Samson* leaves as described^18^. Three days after infiltration, the agroinfiltrated areas were inoculated with a suspension of *Xcc* (10^8^ CFU/mL). Plant responses were checked for 1–3 dpi. The ability of *Xcc* to elicit hypersensitive response (HR) on leaves was assessed by measuring the size of the necrotic lesions developed by *Xcc*.

### Statistical analysis

Statistical analysis was performed using GraphPad Prism 8.0 (GraphPad Software, CA, USA). Data were checked for normality using Shapiro–Wilk’s test and statistically analyzed using one-way analysis of variance (ANOVA). Fisher's Least Significant Difference (LSD) test assessed the differences between means at *P* ≤ 0.05. The data were presented as mean ± standard deviation (SD) for at least three independent experiments.

### Statement of compliance

Permission to collect lime plant samples was obtained from the collection curator of each region before sampling. This study complies with relevant institutional, national, and international guidelines and legislation.

### Supplementary Information

Supplementary Figures.

## Data Availability

All data generated or analyzed that support the findings of this study are available from the corresponding author (H.R), upon reasonable request.
