# Supplementary material for: Transient expression of anti-HrpE scFv antibody reduces the hypersensitive response in non-host plant against bacterial phytopathogen Xanthomonas citri subsp. citri
Source: Sci Rep. 2024 Mar 26;14:7121. doi: 10.1038/s41598-024-57355-w (PMC10965896; doi:10.1038/s41598-024-57355-w)
Supplement: Supplementary file 1 — Supplementary Figures. [file 41598_2024_57355_MOESM1_ESM.pdf]

Transient expression of anti-HrpE scFv antibody reduces the hypersensitive response in non-host plant against bacterial phytopathogen *Xanthomonas citri* subsp. *citri*

Hamideh Raeisi *[1]. Mohammad Reza Safarnejad [2]. Seyed Mehdi Alavi [3]. Maxuel de Oliveira Andrade [4], Naser Farrokhi [5]. Seyed Ali Elahinia [6]

[1]Foodborne and Waterborne Diseases Research Center, Research Institute for Gastroenterology and Liver Diseases, Shahid Beheshti University of Medical Sciences, Tehran, Iran

[2]Department of Plant Viruses, Iranian Research Institute of Plant Protection, Agricultural Research Education and Extension Organization of Iran, Tehran, Iran

[3]Department of Plant Biotechnology, National Institute of Genetic Engineering and Biotechnology, Tehran, Iran

[4]Brazilian Biorenewables National Laboratory (LNBR), Brazilian Center for Research in Energy and Materials (CNPEM), Campinas, Brazil

[5]Departement of Cell & Molecular Biology, Faculty of Life Sciences & Biotechnology, Shahid Beheshti University G.C, Evin, Tehran, Iran

[6]Department of Plant Protection, College of Agricultural Sciences, Guilan University, Rasht, Iran

*__Correspondence:__

Hamideh Raeisi, PhD

ORCID: https://orcid.org/0000-0001-8334-4180

Foodborne and Waterborne Diseases Research Center, Research Institute for Gastroenterology and Liver Diseases, Shahid Beheshti University of Medical Sciences, Shahid Arabi Ave., Yemen St., Velenjak, Tehran, Iran.

Emails: ha.raeesi@gmail.com

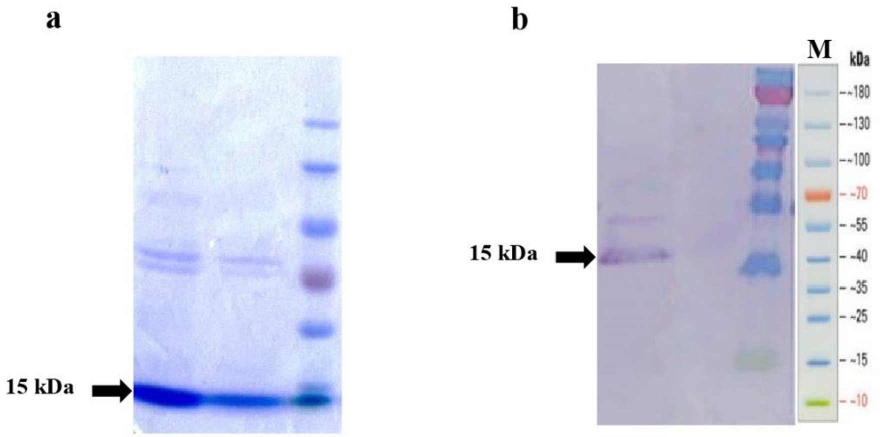

Supplementary Figure 1. (a) SDS-PAGE analysis of the expressed recombinant HrpE (rHrpE) gene in *Escherichia coli* Rosetta strain (DE3). Proteins were separated on 12% polyacrilamide gels and stained with Coomassie brilliant blue. **(b)** Western blot analysis to assess rHrpE purification with dilution 1:10000 of anti-His tag antibody. M: Marker PageRuler Prestained Protein Ladder (Thermo Scientific, USA).

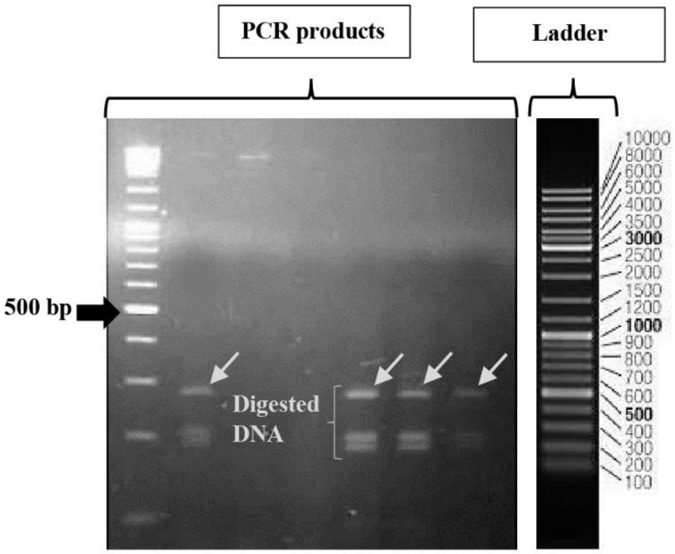

Supplementary Figure 2. Finger printing analysis of PCR product of selected monoclonal scFvs from Tomlinson I phage display library against recombinant HrpE (rHrpE) using *BstN*I digestion. Ladder: GeneRuler DNA Ladder Mix (Thermo Scientific, USA).

a

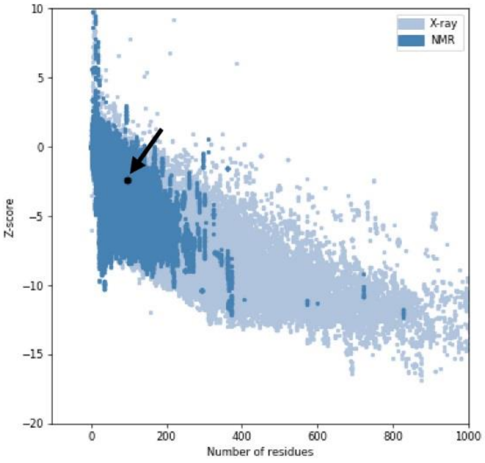

b

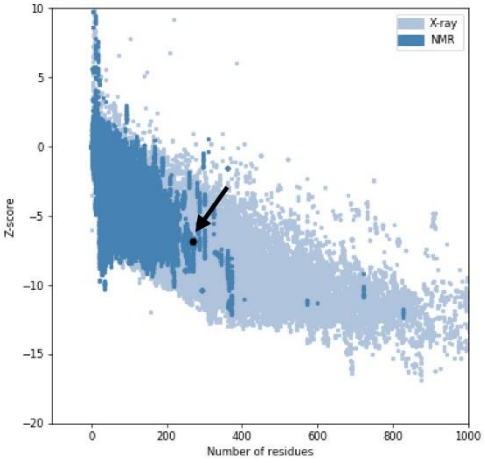

Supplementary Figure 3. ProSA Z-score plot of three-dimensional (3D) modeled **(a)** HrpE and **(b)** scFvH6. The bold black circle shows the position of each model among experimentally solved protein structures.

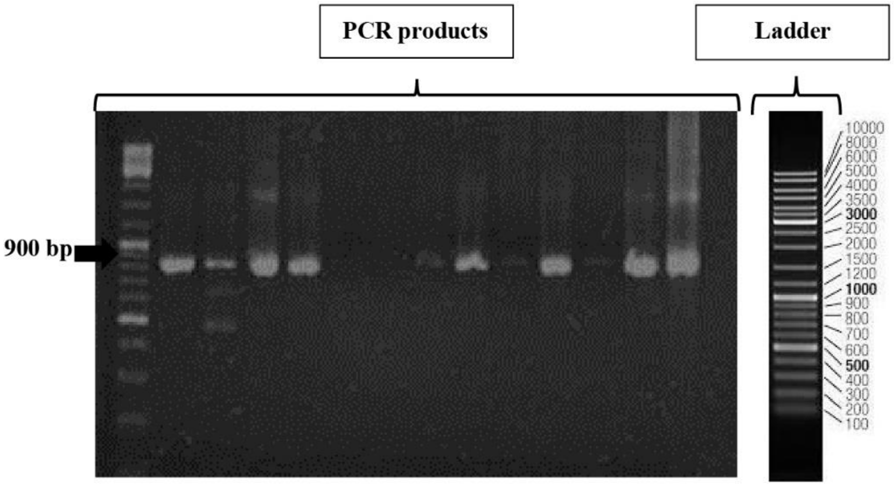

Supplementary Figure 4. PCR amplification of scFvs cDNA in *Agrobacterium radiobacter* transformed with pCAMBIA-scFvH6 using specific scFv primers. Ladder: GeneRuler DNA Ladder Mix (Thermo Scientific, USA).

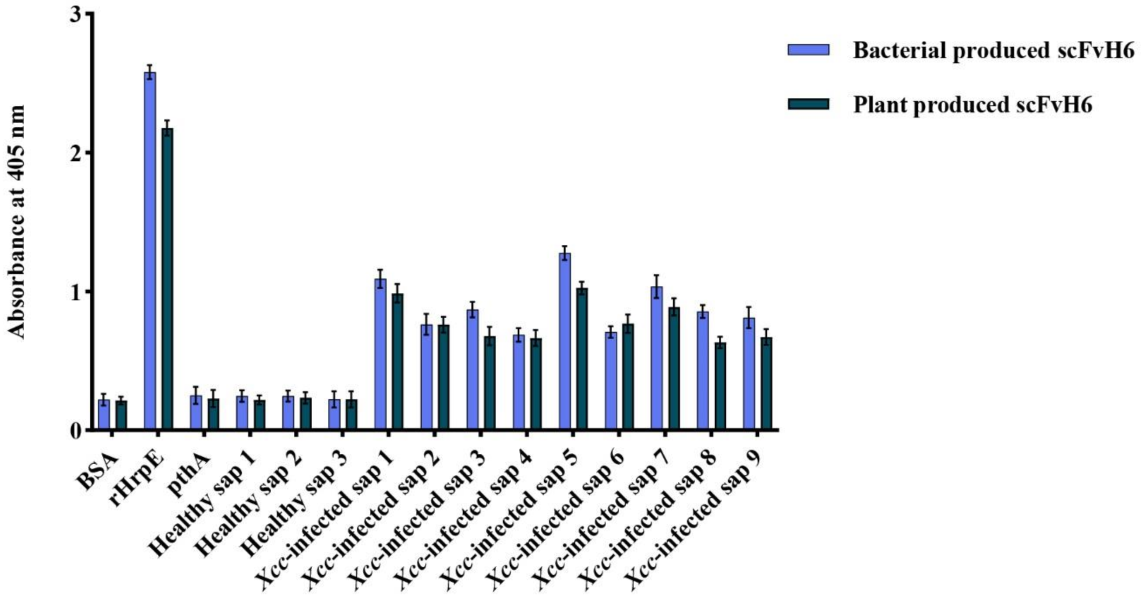

Supplementary Figure 5. The characterization of *in vitro* binding activity of bacterial-produced and plant-produced scFvH6 against recombinant HrpE (rHrpE) and native HrpE in *Xcc*-infected samples using indirect ELISA. Data shown are means ± SD of three independent experiments.

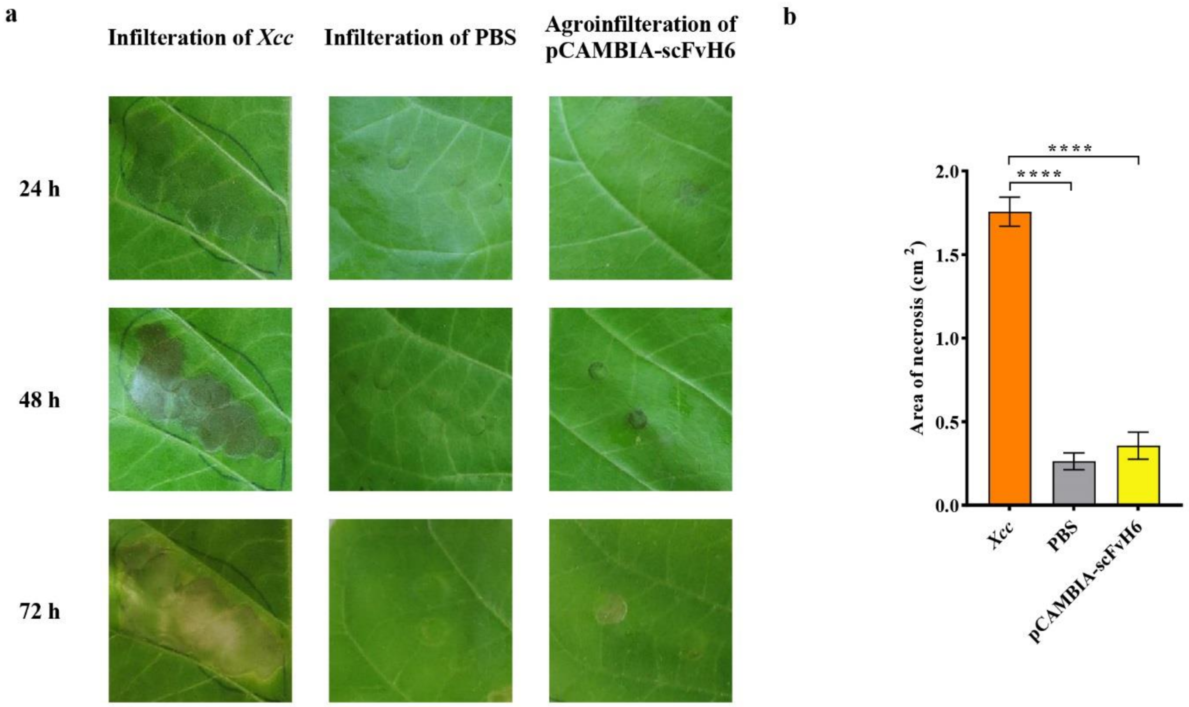

Supplementary Figure 6. (A) Appearance of untransformed *N. tabacum* leaves inoculated with suspension of *Xanthomonas citri* subsp. *citri* (*Xcc*) (10^8 CFU/mL). The leaves were infiltrated agrobacterium bearing pCAMBIA-scFvH6 and PBS were used as negative controls. (B) Comparison of necrosis area in terms of mean lesion size (2 dpi). Data shown are means ± SD of the average lesion size of three independent experiments with six plants in each replicate. *P* value of <0.05 was considered significant (*****P* <0.0001) by unpaired student's t test and one-way ANOVA statistical analysis.

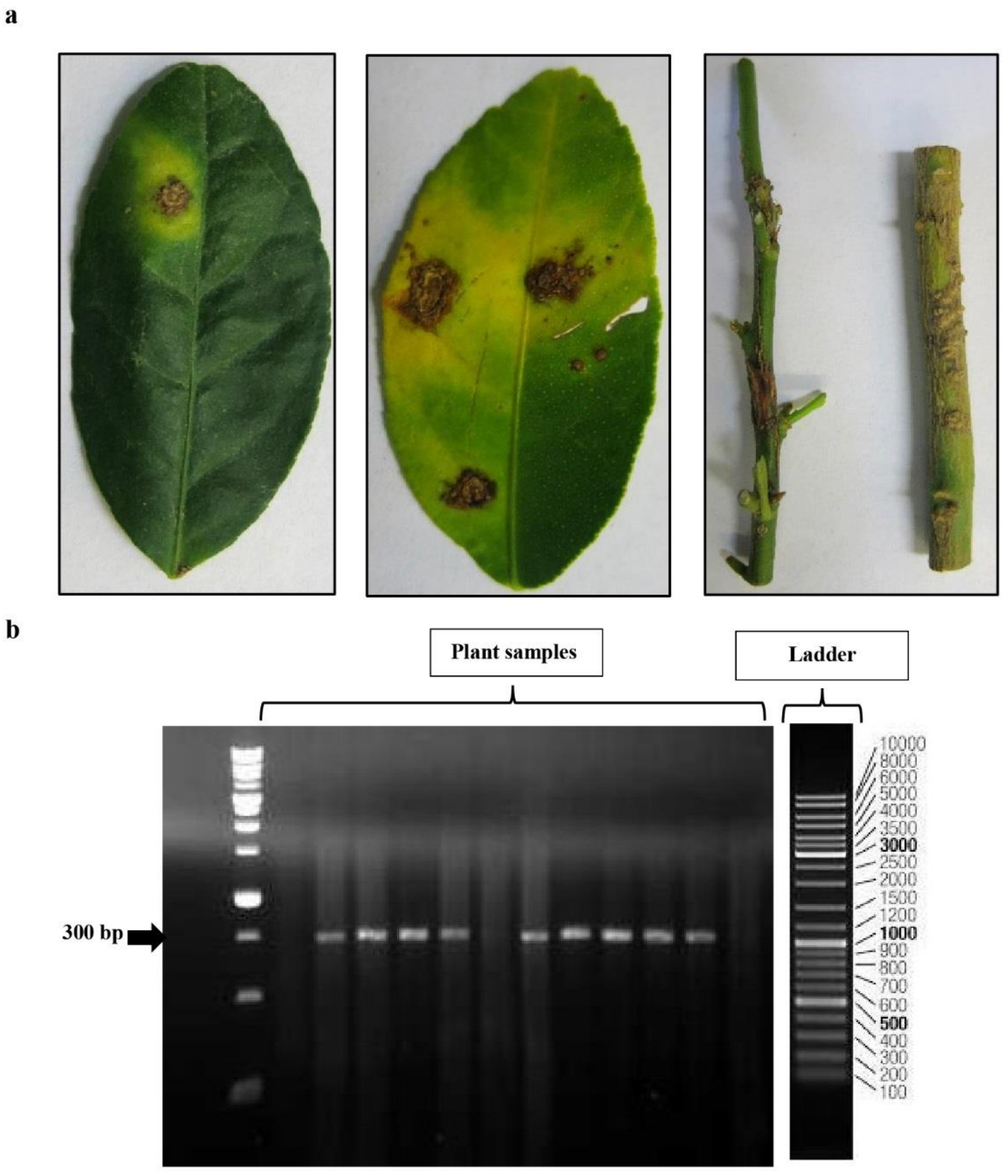

Supplementary Figure 7. (a) Canker symptom, including brown and corky round spots sunken in center with water-soaked margins surrounded by yellow chlorotic halos, in collected citrus samples. **(b)** PCR Amplification *of Xanthomonas citri* subsp. *citri* (*Xcc*) using HrpE-specific primers. Ladder: GeneRuler DNA Ladder Mix (Thermo Scientific, USA).

Original uncropped Western blotting images

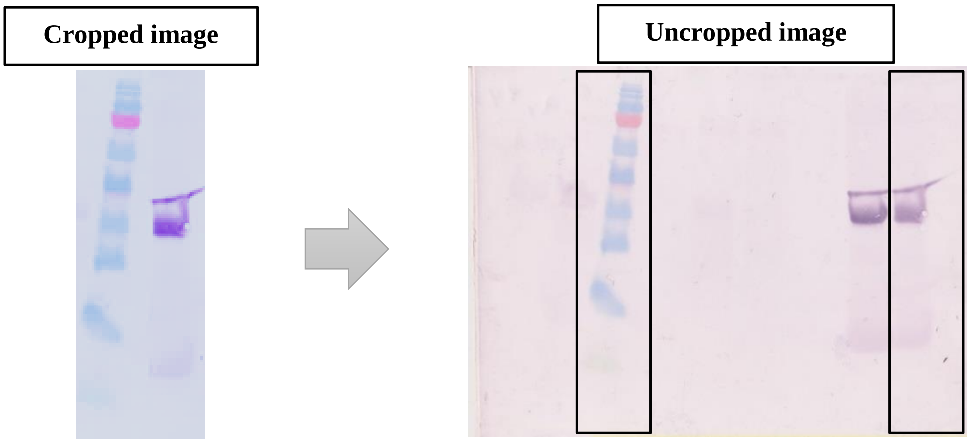

Figure 2. Western blot analysis to assay purification of scFH6 by using anti-His tag antibody (1:10000).

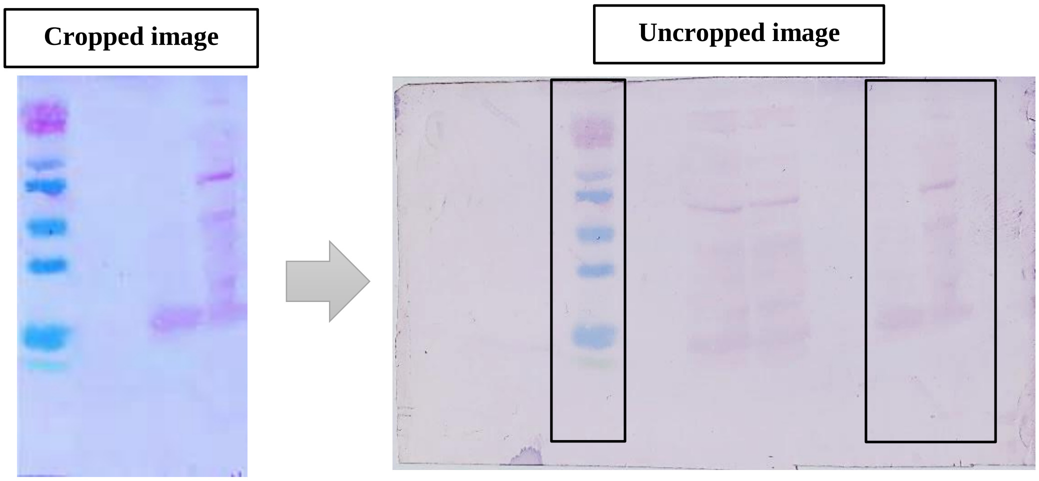

Figure 3. The characterization of *in vitro* binding activity of scFvH6 against rHrpE and native HrpE in *Xcc*-infected samples using Western blot analysis.

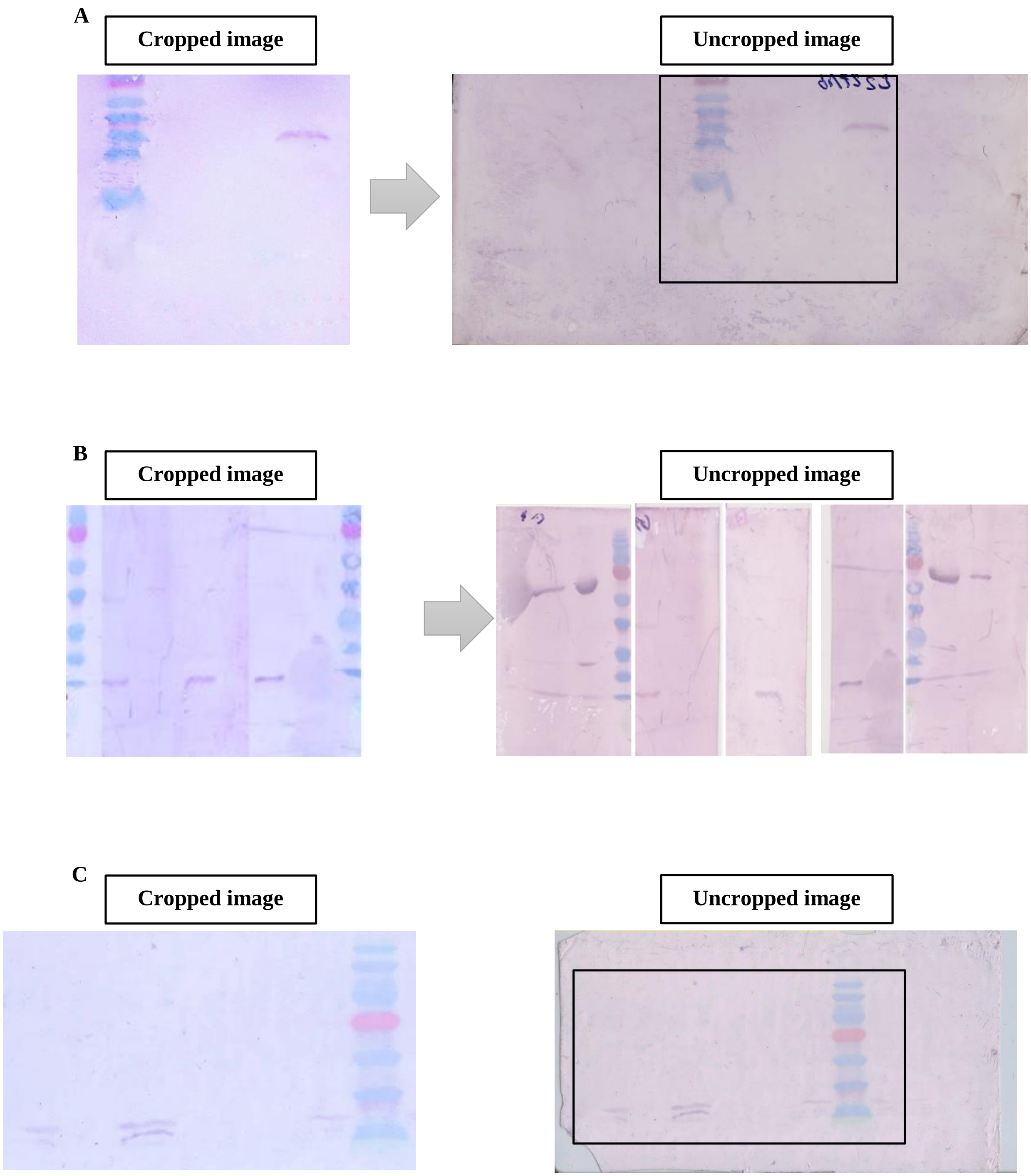

Figure 5. (a) The detection and characterization of expression of scFvH6 in leaves of *Nicotiana tabacum* cv. *Samson* during 1-3 day post inoculation (dpi) using Western blotting. **(b)** The binding activity of plant-produced scFvH6 against rHrpE and **(c)** native HrpE in *Xcc*-infected samples using Western blotting.
